# Factors Related to Favorable Outcomes in Older Adults Undergoing Home-Visit Rehabilitation Therapy

**DOI:** 10.7759/cureus.53740

**Published:** 2024-02-06

**Authors:** Yuji Nakagawa, Akio Goda, Kazushi Fujii, Rua Shimazaki, Naoki Orita, Daisuke Yoshino, Toru Kawara, Masayuki Sakurai, Yoshihiro Ito, Yoshinori Maki

**Affiliations:** 1 Rehabilitation, Hikari Hospital, Otsu, JPN; 2 Physical Therapy, Faculty of Health and Medical Sciences, Hokuriku University, Kanazawa, JPN; 3 Neurosurgery, Hikone Chuo Hospital, Hikone, JPN

**Keywords:** long term care, frenchay activities index, barthel index, elderly, home visit rehabilitation

## Abstract

Background: Increasing elderly population is a major health concern worldwide, requiring various at-home care services. The aim of home-visit rehabilitation therapy is to support at-home living of the elderly and to promote their participation in social activities. There is a paucity of data about the clinical conditions of this population that can contribute to the achievement of goals in-home visit rehabilitation therapy.

Aim: This study aimed to clarify clinical variables that could be related to the achievement of goals in-home visit rehabilitation therapy.

Methods: We collected retrospective clinical data of the older adults who underwent home-visit rehabilitation therapy between July 2006 and June 2021. We searched the clinical variables of home-visit rehabilitation therapy users and their frequency of utilization of home-visit rehabilitation therapy services from the clinical record. The initial and final clinical variables evaluated in this study included the abilities of daily living, degree of being bedridden, dementia rating, and levels of support or long-term care. Those variables were evaluated by rehabilitation therapists and doctors. The users were divided into three groups according to the reason for terminating rehabilitation therapy: goal achievement (achieved group), aggravation of underlying disease (aggravated group), and treatment suspension because of their own/others’ wish (suspended group). The clinical parameters concerning the rehabilitation program, care level, and activities of daily living were evaluated among the groups. The clinical parameters concerning the rehabilitation program, care level, and activities of daily living were statistically evaluated among those three groups, using the chi-square test and Kruskal-Wallis test.

Results: In the achieved, aggravated, and suspended groups, 45, 190, and 38 users were respectively enrolled. The aggravated group showed significantly higher final care level (p = 0.002), degree of being bedridden (p=0.001), and dementia rating (p = 0.017) and significantly lower Barthel index scores (p < 0.001) and Frenchay Activities Index scores (p = 0.001) than the achieved group. Persons requesting the therapy were significantly older adults themselves in the achieved group (p = 0.018). The therapy was significantly performed more than once per week in the achieved group (p = 0.018).

Conclusions: Older adults undergoing self-motivated home-visit rehabilitation therapy more than once per week may contribute to the achievement of the goal.

## Introduction

The increasing proportion of the older population is a major health concern worldwide, including in Japan [1]. Various medical or nursing services are covered by public long-term care insurance, initiated in 2000 to support older adults desiring care at home [1,2]. Home-visit rehabilitation therapy is an option provided by medical or nursing services to improve or maintain the physical and cognitive performance of older adults who cannot attend an outpatient clinic. The expense of home-visit rehabilitation therapy is covered by medical or long-term care insurance [3,4]. Home-visit rehabilitation therapy provides various training programs, including physical, occupational, and speech and swallowing therapy [4]. Setting a definite goal at the initiation of home-visit rehabilitation therapy is mandatory for optimal utilization of the services provided. Termination of home-visit rehabilitation therapy after achieving the goal is considered a favorable outcome. However, in daily clinical situations, one encounters the following types of older home-visit rehabilitation therapy participants: those who achieve the therapy goal, those who cannot continue the therapy because of aggravation of underlying diseases, and those who suspend the therapy because of their own/others’ wishes.

The outcomes of home-visit rehabilitation therapy on the physical performance of older adults, namely, the risk of falling at home, activities of daily living, and general health, have been reported previously [5-9]. However, data concerning the clinical variables that can contribute to achieving the therapy goal among older adults are lacking, especially in Japanese society. Hence, in the present study, we aimed to elucidate the clinical variables possibly associated with achieving the goal of home-visit rehabilitation therapy.

## Materials and methods

Ethical statements

This study was approved by the ethical committee of Hikari Hospital (approval number: 2021-4-2). The requirement for informed consent was waived owing to the retrospective nature of the study and the use of anonymous clinical data.

Study design

We retrospectively collected the clinical data of older adults who underwent home-visit rehabilitation therapy between July 2006 and June 2021 in a single center in Japan. Those patients who underwent and terminated rehabilitation therapy in the aforementioned period were enrolled in this study. Those users whose clinical data was not completely collected were excluded from this study. The individuals were divided into three groups: those who terminated the therapy upon achieving their initial goal (achieved group), those who did not continue the therapy because of aggravation of related underlying diseases (aggravated group), and those who suspended the therapy because of their own or family members' /care managers' wishes (suspended group). We collected clinical variable details at the initiation and termination of the therapy and analyzed differences in clinical variables among the three groups.

Outcomes measure

As exploratory outcomes, the following clinical variables of older home-visit rehabilitation therapy participants which could have been related to the achievement of rehabilitation therapy goals were collected: age; sex; underlying disease requiring therapy (motor system disorder/cerebrovascular disease/other disease); insurance coverage for the therapy (nursing insurance/medical insurance); follow-up hospitalization (other institutes/our institute), period of therapy use (days); frequency of therapy per week (once/twice or more); initial and final Barthel index (BI) scores [10]; initial and final Frenchay Activities Index (FAI) scores [11] to evaluate instrumental activities of daily living; initial and final levels of support or long-term care needed [1-5]; initial and final levels of being bedridden [4]; initial and final dementia ratings; time of home-visit rehabilitation therapy initiation (period during stay at home/period during stay at a facility for older adults/immediately after discharge from a hospital); participants requiring home-visit rehabilitation therapy (older adult/others, such as family members or care managers); and presence of housemates.

Barthel index and Frenchay Activities Index

The BI [10] has 10 variables: feeding, bathing, grooming, dressing, bowel control, bladder control, toilet use, transfer, mobility, and stair use. Considering patients’ ability to perform daily activities, each variable was evaluated using scores of 0, 5, 10, or 15. The minimum and maximum BI scores are 0 and 100, respectively. Rehabilitation therapists evaluated and scored the activities of the users using this battery.

The FAI [11] consists of 15 items: preparing main meals, washing up after meals, washing clothes, light housework, heavy housework, local shopping, social occasions, walking for more than 15 minutes outside, actively pursuing a hobby, driving a car/bus travel, travel outings/car rides, gardening, household/car maintenance, reading books, and gainful work. All variables reflecting instrumental activities of daily living were rated as 0, 1, 2, or 3 according to activity frequency. The minimum and maximum FAI scores are 0 and 45, respectively. Rehabilitation therapists interviewed the users or their major caregivers. In case users were not able to appropriately reply to the interview because of dementia, their caregivers answered. Therapists scored based on the caregivers’ answers.

Levels of support or long-term care needed

Long-term care insurance covers the home visiting service utilized by older adults with support or long-term care needed in Japan [4]. The support and long-term care needed are classified into 2 and 5, respectively. These certifications are determined by two surveys: (a) a doctor examines the physical and mental status of patients and fulfills a document, and (b) the document is evaluated by the certification committee of needed long-term care attended by medical, health, and nursing professionals. A member of the committee visits patients again and interviews them and their caregivers.

Degree of being bedridden and dementia rating

The conditions of older participants of home-visit rehabilitation therapy are classified as grades J, A, B, or C, with each grade having two sub-categories. Older adults who have some form of disability but are mostly independent in their daily life and can go out of the home unaccompanied are categorized as having a grade J condition. Those who can travel using public transport facilities are categorized as grade J1, whereas those who visit neighbors without public transport are categorized as grade J2. The older adults, who are almost independent in their daily life indoors but cannot go outside without support, are categorized as grade A. Grade A1 individuals are those who go out with support and do not stay in bed most of the day. Those who are categorized as A2 do not go out as much and stay in and out of bed during the daytime. Older adults who require some sort of care for indoor daily life and stay in bed most of the time but can maintain a sitting position are categorized as having a grade B condition. Those with a B1 condition can feed themselves and use the toilet using a wheelchair, and those with a B2 condition require support to get into a wheelchair. The older people who stay in bed all the time and require support for toilet use, feeding, and changing clothes are categorized as grade C. Those having a C1 condition can roll over by themselves, and those who cannot are categorized as C2.

The older participants of home-visit rehabilitation therapy with dementia are classified as having grades I, II, III, IV, or M conditions. Subdivisions exist only for grades II and III. Those who are categorized as having a grade I condition have some type of dementia but are mostly independent in daily domestic and social activities. Persons with a grade II condition have symptoms, behavioral issues, or difficulty in communication that interferes with daily life to some degree but can live independently if there is a caretaker. The symptoms are often observed outside the home in older adults classified as having a grade IIa condition, whereas the symptoms can be observed at home as well in those classified as having a grade IIb condition. Those who are categorized as having a grade III condition have occasional symptoms, behavioral issues, or difficulty in communication that interferes with their daily life and require care. The symptoms can be observed during the day and nighttime in those with grade IIIa and IIIb conditions, respectively. Those who are categorized as having a grade IV condition have frequent symptoms, behavioral issues, or difficulty in communication that interferes with their daily life and require constant care. Those who are categorized as having a grade M condition have significant psychotic manifestations, behavioral problems, or severe physical diseases and require specialized medical care. The degree of being bedridden and the dementia rating of the users were evaluated by the doctors.

Statistical analysis

The collected items among the achieved, aggravated, and suspended groups were statistically analyzed. The chi-square test (post hoc analysis: Fisher’s exact test with Holm correction) was utilized for sex, insurance used for home-visit rehabilitation therapy, underlying disease, follow-up hospital, frequency per week initiation timing, participant requesting home-visit rehabilitation therapy, and existence of housemates. Meanwhile, the Kruskal-Wallis test (post hoc analysis: Bonferroni correction) was used for age, support/long-term care needed, degree of bedridden level,dementia rating, and initial and final BI and FAI scores. All statistical analyses were performed using the Statistical Package for Social Sciences software v. 26 (IBM Corp., Armonk, NY), and the statistical significance level was defined at p < 0.05.

## Results

A total of 237 older home-visit rehabilitation therapy participants were enrolled in this study. The numbers in the achieved, aggravated, and suspended groups were 45 (men:women = 19:26), 190 (men:women = 91:99), and 38 (men:women = 11:27), respectively. The mean age of the achieved, aggravated, and suspended groups was 76.98, 79.85, and 80.37 years, respectively (Table 1).

Kruskal-Wallis test (post hoc analysis: Bonferroni correction)

Table 1 shows the analysis with significant differences in the following parameters: higher initial levels of support/long-term care required in the aggravated group compared with the suspended group (p = 0.036); higher final levels of support/long-term care required in the aggravated group than in the achieved and suspended groups (p = 0.002); higher initial and final degree of being bedridden in the aggravated group compared with the achieved group (p = 0.034 and 0.001, respectively); and higher final dementia rating in the aggravated group compared with the achieved group (p = 0.017). The analysis (Table 2) showed that the aggravated group had a significantly lower final BI score than the achievement group (p < 0.001) and a significantly lower final FAI score than the achieved and suspended groups (p = 0.001).

**Table 1:** Analysis of the three groups of older adults undergoing home-visit rehabilitation therapy (Part 1) Mean ± standard deviation (minimum score–maximum score) HVRT: home-visit rehabilitation therapy, NI: nursing insurance, MI: medical insurance Kruskal–Wallis test (post hoc analysis: Bonferroni correction), *: Chi-squared test (post hoc analysis: Fisher’s exact test with Holm correction) ^a^: Ordinal rank of initial and final level of support/long-term care needed: 1: support 1, 2: support 2, 3: long-term care 1, 4: long-term care 2, 5: long-term care 3, 6: long-term care 4, 7: long-term care 5 ^b^: Ordinal rank of initial and final degree of bedridden level: 0: independent, 1: J1, 2: J2, 3: A1, 4: A2, 5: B1, 6: B2, 7: C1, 8: C2 ^c^: Ordinal rank of initial and final dementia rating: 0: independent, 1: I, 2: IIa, 3: IIb, 4: IIIa, 5: IIIb, 6: IV, 7: M ^†^: Achieved group vs. Aggravated group; *p *< 0.05 ^¶^: Aggravated group vs. Suspended group; *p* < 0.05

Variables	Achieved group (*n* = 45)	Aggravated group (*n* = 190)	Suspended group (*n* = 38)	p-value	Post hoc analysis
Age [years]	76.98 ± 11.37	79.85 ± 10.07	80.37 ± 12.79	0.102	
Sex* (men/women) [n]	(19/26)	(91/99)	(11/27)	0.095	
Insurance used for HVRT* (NI/MI) [n]	(45/0)	(189/1)	(37/1)	0.312	
Initial level of support/long-term care needed^a^	4.28 ± 1.50 (1–7)	4.63 ± 1.63 (1–7)	3.92 ± 1.59 (1–7)	0.036	^¶^
Final level of support/long-term care needed^a^	4.39 ± 1.36 (1–7)	5.00 ± 1.55 (1–7)	4.14 ± 1.67 (1–7)	0.002	^†^^, ^^¶^
Initial degree of bedridden level^b^	3.88 ± 1.54 (1–7)	4.67 ± 1.89 (0–8)	4.25 ± 1.40 (0–6)	0.034	^†^
Final degree of bedridden level^b^	3.73 ± 1.72 (0–7)	4.85 ± 1.91 (0–8)	4.27 ± 1.50 (0–7)	0.001	^†^
Initial dementia rating^c^	1.24 ± 1.65 (0–7)	1.60 ± 1.84 (0–7)	1.75 ± 1.55 (0–7)	0.203	
Final dementia rating^c^	1.20 ± 1.67 (0–7)	1.95 ± 1.93 (0–7)	1.81 ± 1.52 (0–6)	0.017	^†^

**Table 2:** Analysis of the three groups of older adults undergoing home-visit rehabilitation therapy (Part 2) Mean ± standard deviation (minimum score–maximum score) MSD: motor system disorder, CVD: cerebrovascular disease, OD: other disease, BI: Barthel index, FAI: Frenchay Activities Index, HVRT: home-visit rehabilitation therapy, NS: Not Significant Kruskal–Wallis test (post hoc analysis: Bonferroni correction), *: Chi-squared test (post hoc analysis: Fisher’s exact test with Holm correction) ^a^: Definition of Initiation timing: A: period during stay at home, B: period during stay at a facility for older adults, C: immediately after discharge from a hospital, ^†^: Achieved group vs. Aggravated group; *p* < 0.05 ^¶^: Aggravated group vs. Suspended group; *p *< 0.05

	Achieved group (n = 45)	Aggravated group (n = 190)	Suspended group (n = 38)	p-value	Post hoc analysis
Underlying disease* (MSD/CVD/OD) [n]	(19/14/12)	(71/57/62)	(16/7/15)	0.560	
Follow-up hospital* (other institutes/our institute) [n]	(39/6)	(168/21)	(34/3)	0.952	
Period of use of home-visit rehabilitation therapy [days]	384.8 ± 408.2	776.3 ± 962.5	517.4 ± 677.6	0.025	NS
Frequency per week* (once/twice or more) [n]	(29/16)	(130/52)	(34/2)	0.018	^†, ¶^
Initial BI score	73.5 ± 22.5 (0–100)	58.2 ± 33.2 (0–100)	67.2 ± 25.6 (5–100)	0.114	
Final BI score	77.1 ± 22.4 (0–100)	47.8 ± 34.4 (0–100)	63.5 ± 28.2 (5–100)	< 0.001	^†^
Initial FAI score	7.6 ± 9.0 (0–31)	6.7 ± 13.1 (0–32)	11.3 ± 17.9 (0–20)	0.068	
Final FAI score	9.5 ± 9.4 (0–33)	4.9 ± 7.3 (0–31)	7.2 ± 6.2 (0–20)	0.001	^†, ¶^
Initiation timing*^a^ (A/B/C) [n]	(28/2/15)	(129/12/42)	(32/1/4)	0.128	
Participant requesting HVRT* (older participant/others) [n]	(13/16)	(21/93)	(4/16)	0.018	^†^
Existence of housemates* (yes/none) [n]	(32/11)	(127/40)	(28/8)	0.941	

Chi-square test (post hoc analysis: Fisher’s exact test with Holm correction)

Chi-squared test analysis (Table 2) showed that for the candidates requiring in-home rehabilitation, the proportion of older participants was significantly higher in the achievement group than in the aggravated group (p = 0.018). For the frequency of home-visit rehabilitation therapy per week, the proportion of older adults in the achievement group who used the service at least once a week was significantly higher than in the other two groups (aggravated and suspended) (p = 0.025).

## Discussion

In this study, we aimed to identify the clinical variables in older adults that could contribute to achieving their home-visit rehabilitation therapy goal. The activities of daily living evaluated using care level, degree of being bedridden, dementia rating, and BI and FAI scores were significantly better in the achieved group than in the aggravated group. Notably, the older adults in the achieved group requested the therapy. Moreover, the older participants in the achieved group were already undergoing home-visit rehabilitation therapy more than once a week.

The care level was evaluated using levels of support/long-term care required, degree of being bedridden, and the dementia rating. The aggravated group had a significantly higher final level of support/long-term care required, degree of being bedridden, and dementia rating than the achieved group. Ohnuma et al. reported that early intervention of home-visit rehabilitation therapy could improve levels of long-term care [12]. In our study, the older adults requiring support 1 or 2 (mild grades of support or long-term care required) were also included, and there was more room for the level of support/long-term care required to worsen than in the participants in the study by Ohnuma et al. (requiring long term care 1 to 5). Furthermore, the individuals enrolled in the present study were relatively older than those in the study by Ohnuma et al. This difference might explain the deterioration of the levels of support/long-term care (transition from support to long-term care or increase in grade) in the aggravated group in the current study. The negative impact of dementia on the deterioration of the support/long-term care levels required has been reported [13]. As the final dementia rating was significantly more severe in the aggravated group than in the achieved group, progressive dementia could have increased the care level required by the older adults.

Activities of daily living of the older participants of home-visit rehabilitation therapy were determined by the BI and FAI scores. A positive impact of home-visit rehabilitation therapy has been previously reported [14]; the positive effect observed in the present study could have resulted in an improvement in the BI and FAI scores in the achieved group. The older participants in the suspended group were undergoing home-visit rehabilitation therapy for longer periods than those in the achieved group. The lack of an apparent positive effect could have resulted in the cessation of the therapy in the suspended group.

Requesting home-visit rehabilitation therapy for oneself appeared to be an important factor in achieving the set goal. The intention to achieve a goal is defined as “achievement motive.” The achievement motive is essential in rehabilitation therapy [15,16]. In the achieved group, a high percentage of those who requested the use of in-home-visit rehabilitation services were the older adult him/herself, rather than the subject family members or care managers. Therefore, the older adults in the achieved group might have been self-motivated to achieve their goals.

In our study, the home-visit therapy frequency also appeared to be important. Home-visit rehabilitation therapy was performed mostly once per week in the suspended group. Insufficient intervention with the therapy could also have been a factor for suspension. Between the achieved and aggravated groups, significance was also detected in the home-visit rehabilitation therapy frequency. As the initial degree of being bedridden was significantly worse in the aggravated group than in the achieved group, the older adults in the aggravated group might have required other home-visit services, such as home-visit care. This might have resulted in a decreased frequency of home-visit rehabilitation therapy in the aggravated group.

Limitations

First, this retrospective study was restricted to a single institute. The number of enrolled older participants for home-visit rehabilitation therapy was limited. Second, possibly because of the older population enrolled in this study, the number of older adults categorized in the aggravated group was larger than that in the achieved or suspended group. This regard could have resulted in false negative results (type II error). In addition, it was not possible to collect complete data on the clinical variables of the older participants of home-visit rehabilitation therapy. This might have been a source of potential selection bias in this study. Third, we did not evaluate the relationship between clinical outcome and rehabilitation therapy, such as training programs and time in a single session. Fourth, we did not evaluate whether the older participants of home-visit rehabilitation therapy enrolled in this study utilized additional rehabilitation programs. These issues need to be addressed in future studies.

## Conclusions

Home-visit rehabilitation therapy can be effective for self-motivated older adults for rehabilitation training. In these adults, rehabilitation therapy performed more than once per week may be more advantageous.
